# Newcastle disease virus V protein inhibits apoptosis in DF-1 cells by downregulating TXNL1

**DOI:** 10.1186/s13567-018-0599-6

**Published:** 2018-10-05

**Authors:** Caiying Wang, Zhili Chu, Wenkai Liu, Yu Pang, Xiaolong Gao, Qiuxia Tang, Jiangang Ma, Kejia Lu, Fathalrhman E. A. Adam, Ruyi Dang, Sa Xiao, Xinglong Wang, Zengqi Yang

**Affiliations:** 1 College of Veterinary Medicine, Northwest A & F University, Yangling, 712100 Shaanxi China; 2 Department of Preventive Medicine and Public Health, Faculty of Veterinary Science, University of Nyala, P.O Box: 155, Nyala, Sudan

## Abstract

Many viral proteins are related to suppressing apoptosis in target cells and are hence beneficial to viral replication. The V protein of Newcastle disease virus (NDV) is one such protein that plays an important role in inhibiting apoptosis in a species-specific manner. However, to date, there have been no reports clarifying the antiapoptotic mechanisms of the V protein. The present study was undertaken to determine the apoptotic potential of the V protein in a chicken embryo fibroblast cell line (DF-1 cell) and to elucidate its molecular mechanisms of action. Here, a yeast two-hybrid system was used to screen the host proteins that interact with the V protein and identified thioredoxin-like protein 1 (TXNL1) as a potential binding partner. Immuno-colocalization of V protein and TXNL1 protein in DF-1 cells further verified the interaction of the two proteins. Through the overexpression of TXNL1 protein and knockdown of TXNL1 protein in DF-1 cells, the effects of NDV replication and cell apoptosis were examined. Cell apoptosis was detected by flow cytometry. The mRNA and protein expression levels of Bax, Bcl-2 and Caspase-3 were detected by quantitative real-time PCR (Q-PCR) and Western blotting. NDV expression was detected by Q-PCR and plaque assay. The results revealed that the TXNL1 protein induced apoptosis and inhibited NDV replication in DF-1 cells. Furthermore, the Western blot and Q-PCR results suggested that TXNL1 induced cell apoptosis through a pathway involving Bcl-2\Bax and Caspase-3. Finally, this work provides insight into the mechanism by which the V protein inhibits apoptosis.

## Introduction

Newcastle disease (ND) is a severe infectious disease in birds. It is a highly pathogenic disease caused by the Newcastle disease virus (NDV). NDV is a member of the avian paramyxovirus type 1 viruses and is classified in the genus *Avulavirus* of the family Paramyxoviridae [1]. NDV strains have different levels of virulence among different avian species [2] and can be grouped into three pathotypes, namely, the lentogenic strains, the mesogenic strains and the viscerotropic or neurotropic velogenic strains, based upon the severity of the disease [3]. Although NDV is currently effectively controlled by vaccination, it remains a potential threat to commercial or backyard fowl production [4], which is endemic in many developing countries. The disease-free countries are more likely to experience accidental outbreaks [2].

The NDV genome is 15 186 or 15 192 nucleotides long and contains six major genes that encode the structural proteins in the order 3′-NP-P-M-F-HN-L-5′ as well as two nonstructural proteins (V and W) [5]. During P gene transcription, the additional nonstructural (V) protein, which shares a common N terminus with the P gene [6], is produced to help with mRNA editing [7]. In the wild-type virus, the V protein is produced at frequencies of approximately 29% [8].

By generating different NDV strain mutants, it is possible to infer that the V protein functions as a virulence factor [9]. The V protein is closely related with host range restriction, which can efficiently overcome innate host defenses [10]. This protein shows its antagonistic activity toward interferon (IFN) by inhibiting the induction of type I IFN caused by NDV infection. Overexpression of the V protein in DF-1 cells can stably weaken the innate cellular immune system [11]. In particular, the cysteine-rich carboxyl terminus of the V protein can target the STAT1 protein selectively as an IFN antagonist [9]. The V protein of NDV plays a significant role in viral replication and serves as a virulence factor [8]. The V protein of NDV also plays a vital role in host range restriction [12]. Clearly, the V protein is a multifunctional protein.

Successful viral replication requires a proapoptotic mechanism to achieve the efficient spread of progeny; when apoptosis is inhibited by viruses, infected cells are prevented from dying prematurely, thus facilitating viral replication, spread, or persistence [13]. In a previous study, NDV was reported to trigger apoptosis by activating the mitochondrial/intrinsic pathway in tumor cells [14]. NDV has been reported to induce autophagy and apoptosis in chicken cells; hence, inhibition of apoptosis enhances autophagy and promotes NDV replication [15].

The HN gene of NDV and human TNF-α act synergistically to cause apoptosis in the HeLa cell line by upregulating the SAPK/JNK pathway [16, 17]. Furthermore, the V protein plays an important role in preventing apoptosis in a species-specific manner [12]. However, to date, there has been no report clarifying the antiapoptotic mechanisms of the V protein.

In the present study, a yeast two-hybrid (Y2H) screen was performed, and the result indicate that the V protein can interact with thioredoxin-like protein 1 (TXNL1). TXNL1, a member of the thioredoxin family, is a two-domain, 32-kDa protein that contains an N-terminal Trx domain and a C-terminal DUF1000 domain that interacts with the 26S proteasome [18]. A recent study showed that TXNL1 may contribute to cancer metastasis [19]. A previous study revealed that the TXNL1-XRCC1 pathway regulates cisplatin-induced cell death in gastric cancer cell lines [20]. TXNL1 has been reported to induce apoptosis in cisplatin-resistant human gastric cancer cell lines [21]. However, there has been no related report regarding mechanisms by which TXNL1 affects viral replication.

To study the function of the V protein, a series of experiments were designed. The present study revealed a novel and independent role of TXNL1 in regulating apoptosis and NDV replication in DF-1 cells. Moreover, this study contributes to the knowledge of the antiapoptotic mechanism of the V protein.

## Materials and methods

### Yeast two-hybrid screening of protein interactions

With the V protein as the bait protein, the host proteins that interacted with the V protein from a chicken embryo fibroblast (CEF) Y2H library were obtained. Before screening, the bait protein was tested against the autoactivity and toxicity of the V protein. Then, a concentrated Y2H pGBKT7-V culture with a Y2H library aliquot was mixed for mating according to the Matchmaker™ Gold Yeast Two-Hybrid protocol (Clontech, USA). Seventy 150-mm DDO/X/A (double dropout medium supplemented with X-α-Gal and lacking tryptophan and leucine) plates were used to screen the clones after mating for 3–5 days. All blue colonies that appeared on the DDO/X/A plates were then patched out and allowed to grow on QDO/X/A (quadruple dropout medium supplemented with X-α-Gal and lacking adenine, histidine, tryptophan and leucine). To eliminate false positives, the blue colonies were screened twice on QDO/X/A plates to rescue the additional library plasmids. The bait plasmid (pGBKT7-53 or pGBKT7-Lam) was cotransformed into Y2H Gold with the prey plasmid (pGADT7-T) to serve as positive and negative controls, respectively. The rescued genuine positive AD/library VHH inserts were further sequenced and aligned using the NCBI BLASTP program [22].

Previous research showed that two-hybrid screening of yeast identified over a dozen proteins as having potential interactions with NDV V protein. The proteins identified were predicted as being involved in RNA binding, cancer-related pathways, and apoptosis-related pathways. Previously, our group focused on the V protein mediated host apoptosis. Three of them were reported to affect cell apoptosis including TXNL1, CacyBP/SIP and NumB. Compared with another two proteins, TXNL1 was reported more deterministic to participate in apoptosis [20]. We chose it for further study due to its involvement in the cell apoptosis adjustment.

### Construction of a recombination plasmid

The primer sequences for TXNL1 were designed according to the published Gallus TXNL1 (XM_424463.5), as shown in Table 1. TXNL1 was amplified from the CEF, and the cDNA was synthesized using a reverse transcription polymerase chain reaction (RT-PCR) [23]. Then, enzyme digestion and ligation were performed, and the specific fragments were cloned into the pCMV-HA cloning vector (TaKaRa, Dalian, China). Furthermore, for selection, ampicillin was used as a specific antibiotic. The positive clones were verified using PCR and sequencing. Verification was performed by PCR and double restriction enzyme digestion by EcoRI (TaKaRa, Dalian, China) and NotI (TaKaRa, Dalian, China). The V gene cloned into the pCAGEN plasmid with a Flag tag at the N-terminal of the V gene was named pCAGEN-Flag-V (unpublished data).

**Table 1:** **Primers used in this work**

Primer	Primer sequence (5^′^ to 3^′^)
TXNL1-F	CGGAATTCATGGTGGGCGTGAAGCTGAT
TXNL1-R	TTGCGGCCGCTTAGTGGCTCTCTCCTTTTT
Q-TXNL1-F	TCGCTAACGACACCGAGTTC
Q-TXNL1-R	TTCTAACCCCACAGCATCCG
Q-Bcl2-F	CTTCCGTGATGGGGTCAACT
Q-Bcl2-R	AGGTACTCGGTCATCCAGGT
Q-Caspase3-F	CCATGGCGATGAAGGACTCT
Q-Caspase3-R	CCCGCTAGACTTCTGCACTT
Q-FasL-F	GAGGTGTTGACCCACGTTGT
Q-FasL-R	AGTTGATGCGCTTGTCCTCC
Q-Caspase9-F	GCTTGTCCATCCCAGTCCAA
Q-Caspase9-R	CAGTCTGTGGTCGCTCTTGT
Q-NDV-F	TCCCAAGCGCGAGTTACTTT
Q-NDV-R	TTGTTCGCCACGACCATACA
Q-βactin-F	TTACCCACACTGTGCCCATC
Q-βactin-R	GGGCACCTGAACCTCTCATT

### Cell culture and plasmid transfection

The CEF cell line (DF-1) was grown in Dulbecco’s modified Eagle’s medium (DMEM) supplemented with 10% fetal bovine serum (FBS; Gibco;USA), 100 U/mL penicillin, and 0.1 mg/mL streptomycin at 37 °C in a 5% CO_2_ incubator, as described by [24].

DF-1 cells were plated at 5 × 10^4^ per well in a 24-well plate and incubated for 24 h. The cells were co-transfected with equal amounts of pCMV-HA-TXNL1 or pCAGEN-Flag-V and pCMV-HA-TXNL1 using TurboFect Transfection Reagent (Thermo Fisher).

DF-1 cells were plated at 5 × 10^4^ per well in a 24-well plate and incubated for 24 h. The cells were transfected with small interfering RNA (siRNA) using Lipofectamine 2000 (Invitrogen, USA). Briefly, 2 μg of plasmids and 4 μL of transfection reagent in 0.2 mL of Opti-MEM (Gibco) was mixed for 15–20 min at room temperature. The DNA complex was added dropwise to the plate and incubated for 48 h.

### Small interfering RNA (siRNA) assays

RNA interference was used to knock down TXNL1. DF-1 cells were grown to 60–70% confluence in 24-well plates and then transfected with 20 pmol siRNA using 2 μL of Lipofectamine 2000 (Invitrogen) in Opti-MEM medium (Invitrogen). After 24 h, cells were subsequently infected with NDV, as described previously. After an additional 24 h, the cells were harvested for further experiments. The siRNA were designed and synthesized by the Sangon Company (Shanghai, China) with the following sequences: TXNL1-chicken-860 (Sense: GCAUUAUCCAACUUCGCUATT, Antisense: UAGCGAAGUUGGAUAAUGCTT), TXNL1-chicken-432 (Sense: GCGGAUUGACCAGUAUCAATT, Antisense: UUGAUACUGGUCAAUCCGCTT), TXNL1-chicken-290 (Sense: UUGAAAGCUGGAGCUAUCCTT, Antisense: UUGAAAGCUGGAGCUAUCCTT), negative control (NC) (Sense: UUCUCCGAACGUGUCACGUTT, Antisense: ACGUGACACGUUCGGAGAATT).

### Viruses and viral infection of DF-1 cells

One virulent strain was used in this study, namely, F48E9, which is kept in our laboratory. The virus suspension was propagated in 10-day-old specific pathogen-free chicken embryos, and allantoic fluids that exhibited high HA titers were collected and stored at −80 °C until further use [22].

To infect DF-1 cells, the viruses suspended in the culture medium were added to the plate cultures at a multiplicity of infectivity (moi) of 0.1. Cells were incubated with the viruses at 37 °C for 60 min. After incubation, the supernatant was removed, and the cells were washed twice with PBS. Finally, 2% FBS DMEM was added into the culture. The infections were performed in an incubator at 37 °C supplied with 5% CO_2_.

### Viral plaque assay

The viral titer was measured by the plaque assay. Briefly, the confluent monolayer of DF-1 cell growth in a 24-well plate was inoculated with different dilutions of cell supernatant (0.1–10 µL per well). After adsorption for 1 h at 37 °C in 5% CO_2_, the liquid was aspirated, overlaid with DMEM with 2% FBS and 1% methylcellulose and cultured at 37 °C in 5% CO_2_. After 3–5 days, the DMEM was removed and the cells were gently washed three times with PBS and fixed with 4% PFA for 30 min. After airing the plate, the plaques were stained with crystal violet for 20 min. Finally, pictures were taken after the plate was washed with slowly running water.

### Western blot analysis

The cells harvested at the indicated time points were washed three times with PBS and lysed in RIPA buffer (HEART BIOLOGICAL, Shanghai, China) with 1 mM PMSF for 15 min on ice. Equal amounts of cell extracts were prepared and separated using 12% SDS-PAGE. Proteins were transferred to an Immun-Blot PVDF membrane (Immobilon^®^-P), and Western blot analysis was performed according to a standard protocol. The primary antibodies used in this research were as follows: Bcl-2 (1:500; Proteintech; Wuhan; China), Bax (1:400; Boster; Wuhan; China), Caspase-3 (1:400; BIOSS; Beijing; China), TXNL1 (1:500; Proteintech; Wuhan; China), GAPDH (1:2000; Sungene Biotech; Tianjin; China), goat antimouse (1:5000; Sungene Biotech; Tianjin; China), and goat antirabbit (1:5000; Sungene Biotech; Tianjin; China).

### Quantitative real-time PCR (Q-PCR)

The mRNA levels of the NDV, FasLG, Bax, Bcl-2, Caspase-3 and β-actin genes (the sequences are in Table 1) were analyzed by a two-step real-time PCR. Total RNA was prepared from the cells with RNAiso Plus (TaKaRA), and the first-strand cDNA was synthesized using the StarScript II First-Strand cDNA Synthesis Mix (GeneStar) with random primers. Real-time PCR was conducted with the 2 × RealStar Green Fast Mixture (GeneStar) according to the manufacturer’s instructions. Relative expression values were normalized using an internal β-actin control. All results were analyzed using the of 2^−ΔΔCT^ method. Melting curves were analyzed to determine the specificity of each reaction. All reactions were conducted in duplicate for each sample, and the mean was calculated for each gene target.

### Cell immunofluorescence staining

DF-1 cells were plated on confocal dishes, and when the growth reached 50–60%, the cells were transfected with pCAGEN-Flag-V using TurboFect (Thermo, USA). After 24 h, the cell samples were directly fixed in 4% PFA in PBS for 15 min and in 0.5% Triton X-100 in PBS for 10 min. The cell samples were blocked in 1% BSA at room temperature for 30 min and then exposed to the primary antibody Flag (1:1000; Thermo; USA), TXNL1 (1:300; Proteintech; Wuhan; China) overnight at 4 °C. Then, the samples were washed three times with PBS at 5 min intervals and incubated with donkey antimouse IgG H&L Alexa Fluor 594 (Invitrogen, Carlsbad, CA, USA) and goat antirabbit IgG H&L Alexa Fluor 488 (Abcam, Cambridge, UK) at 37 °C for 60 min, followed by three more washes with PBS. The cell nuclei were stained using Hoechst 33342. Finally, images were captured using confocal microscopy (ANDOR Revolution WD confocal).

### Flow cytometric analysis of apoptosis

Phosphatidylserine translocation to the outer leaflet of the plasma membrane was detected using annexin V staining of the transfected cells. The assay was performed using the Apoptosis Assay Kit (Invitrogen, USA) according to the manufacturer’s protocol. First, the transfected DF-1 cells were harvested by trypsinization at 48 h, washed with PBS and resuspended in 1X annexin-binding buffer to a density of 1 × 10^6^ cells/mL. The cells were then labeled with 5 μL of Alexa Fluor^®^ 488 annexin V and 1 μL of the 100 μg/mL PI working solution and incubated at room temperature for 15 min in the dark. Thereafter, 400 μL of 1X annexin-binding buffer was added, and the cells were kept on ice and analyzed by flow cytometry (BD LSRFortessa, USA).

### Statistical analysis

The significant differences in serological analysis among different groups were statistically evaluated using Student’s *t* test with Prism 5.0 (Graph Pad Software, Inc., San Diego, CA, USA). Calculated *P* values of < 0.05 were considered significantly different.

## Results

### V protein interacts with and downregulates the expression of TXNL1 in DF-1 cells

**Figure 1:**
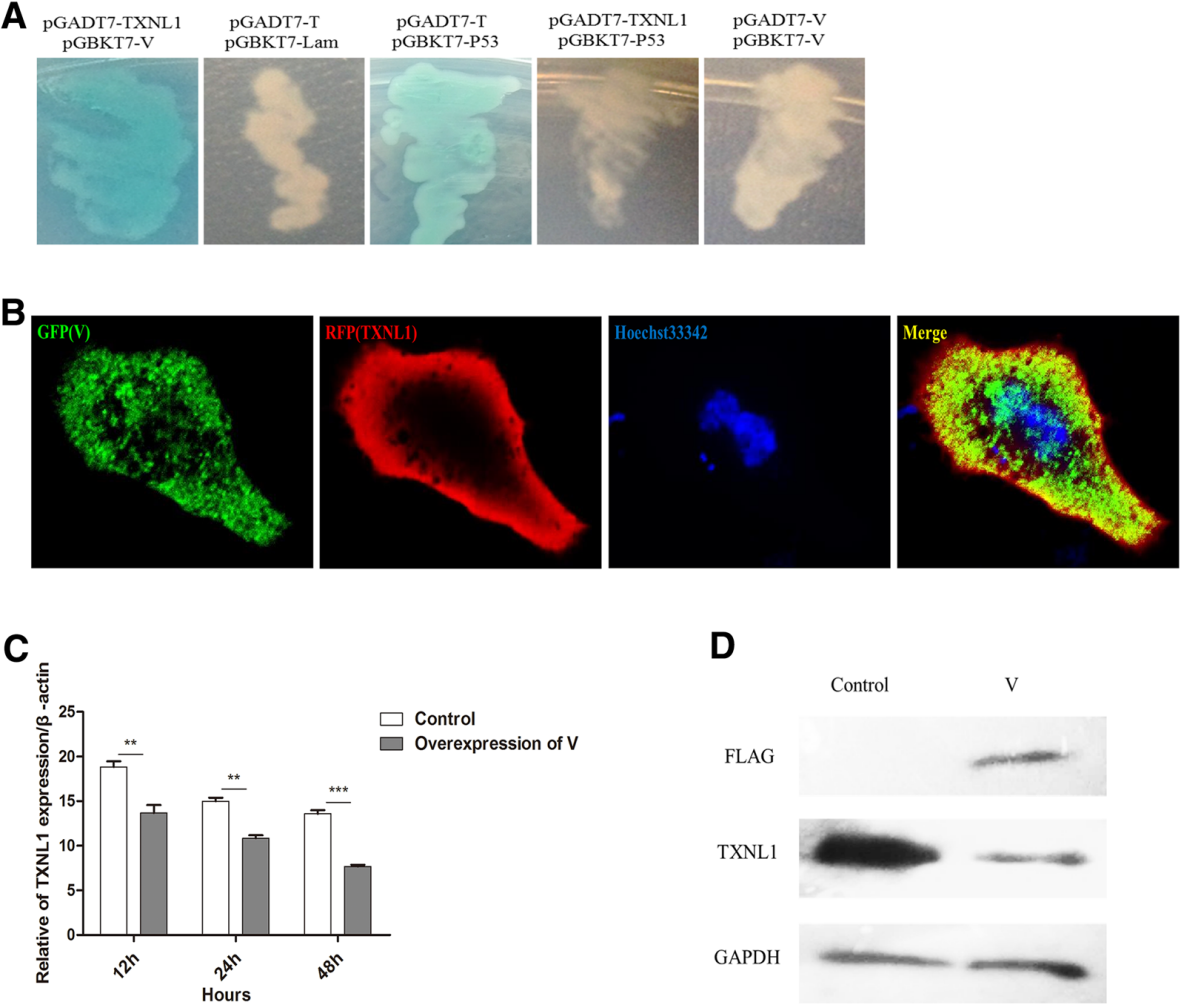
**V protein interacts with TXNL1 and downregulates the expression of TXNL1. A** Interaction between V and TXNL1 in the yeast system. The white clones show that the bait (pBD-V) had no autoactivator activity in yeast. When pGBKT7-V and pGADT7-TXNL1 were cotransformed into the Y2H Gold yeast strain, the cotransformants turned blue on DDO/X/A and QDO/X/A plates after incubation. At the same time, the pGBKT7-p53 and pGADT7-T cotransformants were used as positive controls, whereas the pGBKT7-Lam and pGADT7-T cotransformants were used as NC. **B** Confocal analysis of TXNL1 and V in DF-1 cells. V protein was transfected into DF-1 cells and assessed by immunofluorescence staining. V protein was detected with goat antimouse monoclonal antibody (MAb) and visualized with Alexa Fluor 488 (green). TXNL1 was detected with a rabbit anti-TXNL1 polyclonal antibody and visualized with Alexa Fluor 546 (red). Yellow indicates colocalization of the V protein and TXNL1 in the merged image **C** V protein downregulated the expression of TXNL1. DF-1 cells were transfected with V and control plasmids (pCAGEN) for 48 h. The RNA was extracted according to the method previously described. The mRNA levels of endogenous TXNL1 were detected using Q-PCR. **D** DF-1 cells were transfected with V and control plasmids (pCAGEN) for 48 h. Whole-cell extracts were prepared for Western blot analysis that was specific for the indicated proteins.

The Y2H system was used to identify host proteins that interact with the V protein. Our previous work has shown that 15 proteins have the potential to interact with the V protein [25]. In this work, with further screening, the CEF yeast library was used with the V protein as a bait, we identified another 6 proteins including TXNL1 (Table 2). Further TXNL1 was cloned into the pGADT7 vector, and the pGADT7-TXNL1 and pGBKT7-V cotransformants were positive on DDO/X/A and QDO/X/A plates after incubation (Figure 1A), which indicates that the V protein and TXNL1 have an interactive relationship. The localization of the V protein and TXNL1 was examined using immunofluorescence and confocal microscopy in DF-1 cells transfected with pCAGEN-Flag-V. As can be clearly seen from the picture, the V protein (GFP) and TXNL1 (RFP) colocalized in the cytoplasmic region (Figure 1B). To explore the correlativity between the V protein and TXNL1, the expression of endogenous TXNL1 at the mRNA and protein levels was detected after the transfection of pCAGEN-Flag-V in DF-1 cells; the results suggest that the expression of endogenous TXNL1 in these cells was dramatically lower than in the control (pCAGEN) (Figures 1C and D). The result suggest that the V protein interacts with TXNL1 and downregulates the expression of TXNL1 in DF-1 cells.

**Table 2:** **Proteins screened in this work**

Protein no.	Protein name	Gene	NCBI protein accession no.	Max identity (%)	No. of clones
1	Calcyclin binding protein (CACYBP)	CacyBP/SIP	XM_422279.6	99	4
2	Rho GDP dissociation inhibitor gamma	ARHGD1G	XM_003642163.4	100	2
3	Citrate synthase	CS	XM_015300289.2	100	2
4	adenylate kinase 3	AK3	XM_015280190.2	99	1
5	Uncharacterized LOC107049257	LOC107049257	XM_025145642.1	100	7
6	Filamin A interacting protein 1	FILIP1	XM_015284761.2	99	1
7	Elastin microfibril interfacer 1	EMILIN1	XM_015285012.2	100	1
8	WWC family member 3	WWC3	XM_015273752.2	96	3
9	Sarcoglycan beta	SGCB	NM_001031155.1	97	1
10	B-TFIID TATA-box binding protein associated factor 1	BTAF1	XM_015288828.2	98	1
11	Adenylosuccinate synthase	ADSS	XM_015283953.1	95	3
12	Collagen type VI alpha 3 chain	COL6A3	XM_015289155.2	98	1
13	Frizzled-1	cFz-1	AF224314.1	97	1
14	Tet methylcytosine dioxygenase 1	TET1	XM_025151681.1	95	1
15	Heterogeneous nuclear ribonucleoprotein H2	HNRNPH2	XM_015293725.2	99	1
The information above cited from our preliminary work reported by Chu et al. [32]					
16	Thioredoxin like 1	TXNL1	XM_424463.6	99	2
17	Nudix hydrolase 21	NUDT21	NM_001277674.1	99	2
18	Fatty acid binding protein 7	FABP7	NM_205308.2	99	2
19	Lysyl oxidase-like 1	LOXL1	XM_424060.6	99	1
20	Leucyl-tRNA synthetase	LARS	XM_414663.6	99	1
21	Cysteine rich angiogenic inducer 61	CYR61	NM_001031563.1	97	1

### Overexpression of the V protein in DF-1 cells inhibited apoptosis through the Bcl-2\Bax-Caspase-3 pathway

**Figure 2:**
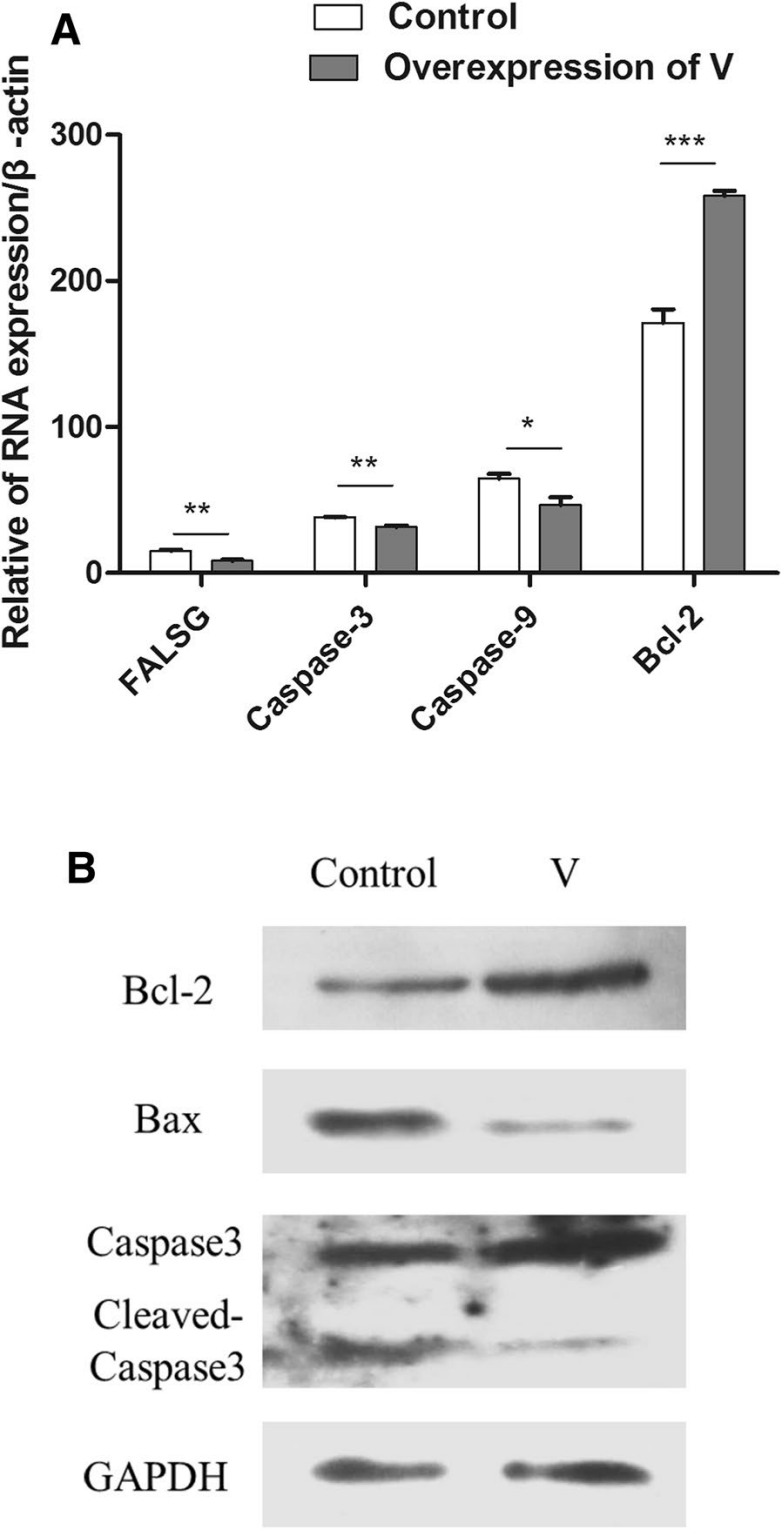
**V protein overexpression in DF-1 cells inhibited apoptosis through the Bcl-2\Bax-Caspase-3 pathway. A** V and control (pCAGEN-flag) plasmids were transfected into DF-1 cells for 48 h before the cells were harvested. The RNA was extracted according to the previously described method. The mRNA levels of some apoptosis-related genes (proapoptosis genes Caspase-3, Caspase-9, and FasLG, and the antiapoptosis gene Bcl-2) were detected using Q-PCR. **B** DF-1 cells were transfected with V and control (pCAGEN-flag) plasmids for 48 h. Whole-cell extracts were prepared for Western blot analysis that was specific for the indicated proteins.

The V protein has been reported to play an important antiapoptotic role [12]. To explore which pathway was involved in the antiapoptosis function of the V protein, a series of apoptosis-related genes were tested at the mRNA level after transfection of the V protein into DF-1 cells for 48 h. These genes included the proapoptotic genes Caspase-3, Caspase-9, and FasLG and the antiapoptotic gene Bcl-2. The result revealed that the proapoptotic genes had significantly higher expression levels in the TXNL1-transfected DF-1 cells than the levels in the control (pCMV-HA), while the antiapoptosis gene had a significantly lower expression level in the transfected cells than that in the control (pCMV-HA) (Figure 2A). The result was also confirmed at the protein level, as can be seen in the picture (Figure 2B). When V protein was overexpressed in DF-1 cells, the expression levels of cleaved Caspase-3 and Bax were significantly lower than those in the control, while the expression of Bcl-2 was higher than that in the control. These results indicate that the V protein inhibits apoptosis through the Bcl-2\Bax-Caspase-3 pathway in DF-1 cells.

### TXNL1 inhibits NDV replication in DF-1 cells

**Figure 3:**
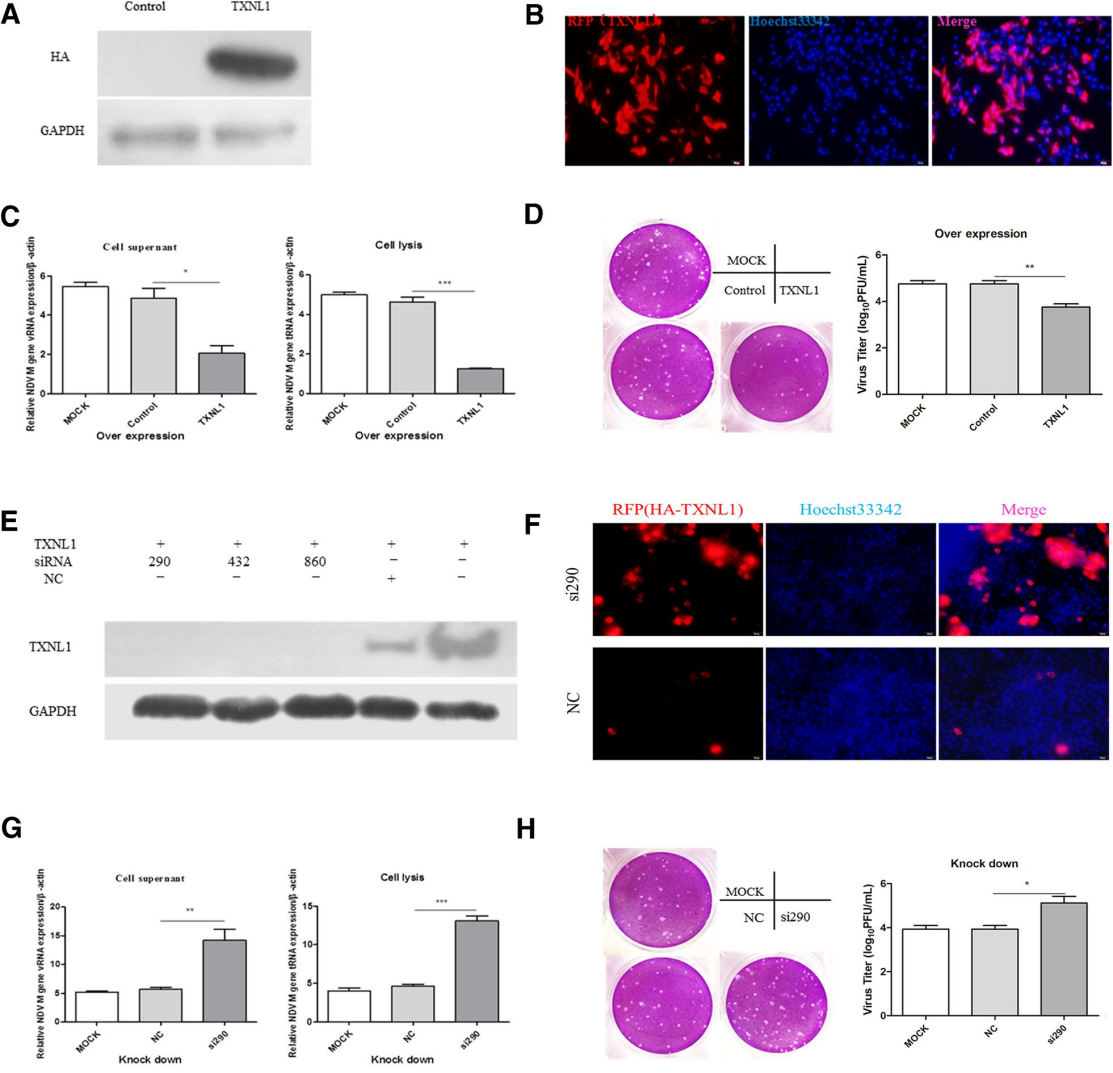
**TXNL1 inhibits NDV replication in DF-1 cells. A** DF-1 cells were transfected with TXNL1 and control (pCMV-HA) plasmids for 24 h and assessed by Western blot. **B** DF-1 cells were transfected with TXNL1 and control (pCMV-HA) plasmids for 24 h and assessed by immunofluorescence staining. **C** DF-1 cells were transfected with TXNL1 and control plasmids for 24 h and infected with F48E9 (0.1 moi) for 24 h. The RNA was extracted according to the previously described method. The mRNA level of NDV was detected using Q-PCR. **D** The viral titer of F48E9 was detected using the viral plaque assay. **E** The efficiencies of the three siRNA were detected by Western blot. **F** The efficiencies of the three siRNA were detected by immunofluorescence staining. **G** DF-1 cells were transfected with si290 or NC for 12 h and infected with F48E9 (0.1 moi) for 24 h. The RNA was extracted according to the previously described method. The mRNA level of NDV was detected using Q-PCR. **H** The viral titer of F48E9 was detected using the viral plaque assay. Three independent experiments were performed, and data are shown as the means ± standard deviations of three replicates from a representative experiment.

Since the V protein plays an important role in viral replication, in order to systematically investigate the relationship between TXNL1 and NDV replication, DF-1 cells were transfected with TXNL1 and control (pCMV-HA) plasmids (Figures 3A and B) for 24 h and then infected with F48E9 (0.1 moi) for an additional 24 h. Q-PCR was performed, and the results revealed that the mRNA levels of viral genomic RNA (vRNA) and total RNA (tRNA) from TXNL1-overexpressed DF-1 cells were significantly decreased when compared with those of the control (pCMV-HA) (Figure 3C). We also confirmed our result by viral plaque assay; as the figure shows, the viral titer of overexpressed TXNL1 was significantly lower than that of the control (pCMV-HA) (Figure 3D). These data indicate that overexpression of TXNL1 could inhibit NDV replication in DF-1 cells.

To further study the effects of TXNL1 on viral replication, three siRNA were designed and synthesized to knockdown endogenous TXNL1. First, the interference efficiency of the synthetic siRNA was evaluated. The figure indicates that all three siRNA functioned; si-TXNL1 (290) was chosen for the subsequent experiments (Figure 3E). We also evaluated the interference efficiency of si290 at the cellular level by immunofluorescence assay (Figure 3F). Q-PCR was performed, and the results indicate that the levels of vRNA and tRNA in the TXNL1-knockdown DF-1 cell were significantly increased compared with those of the NC (Figure 3G). This effect was also confirmed by viral plaque assay; the results revealed that the viral titer of the knockdown group was significantly higher than that of the NC (Figure 3H). These data show that the knockdown of TXNL1 clearly promoted NDV replication in DF-1 cells.

### TXNL1 induces apoptosis in DF-1 cells through the Bcl-2\Bax-Caspase-3 pathway

**Figure 4:**
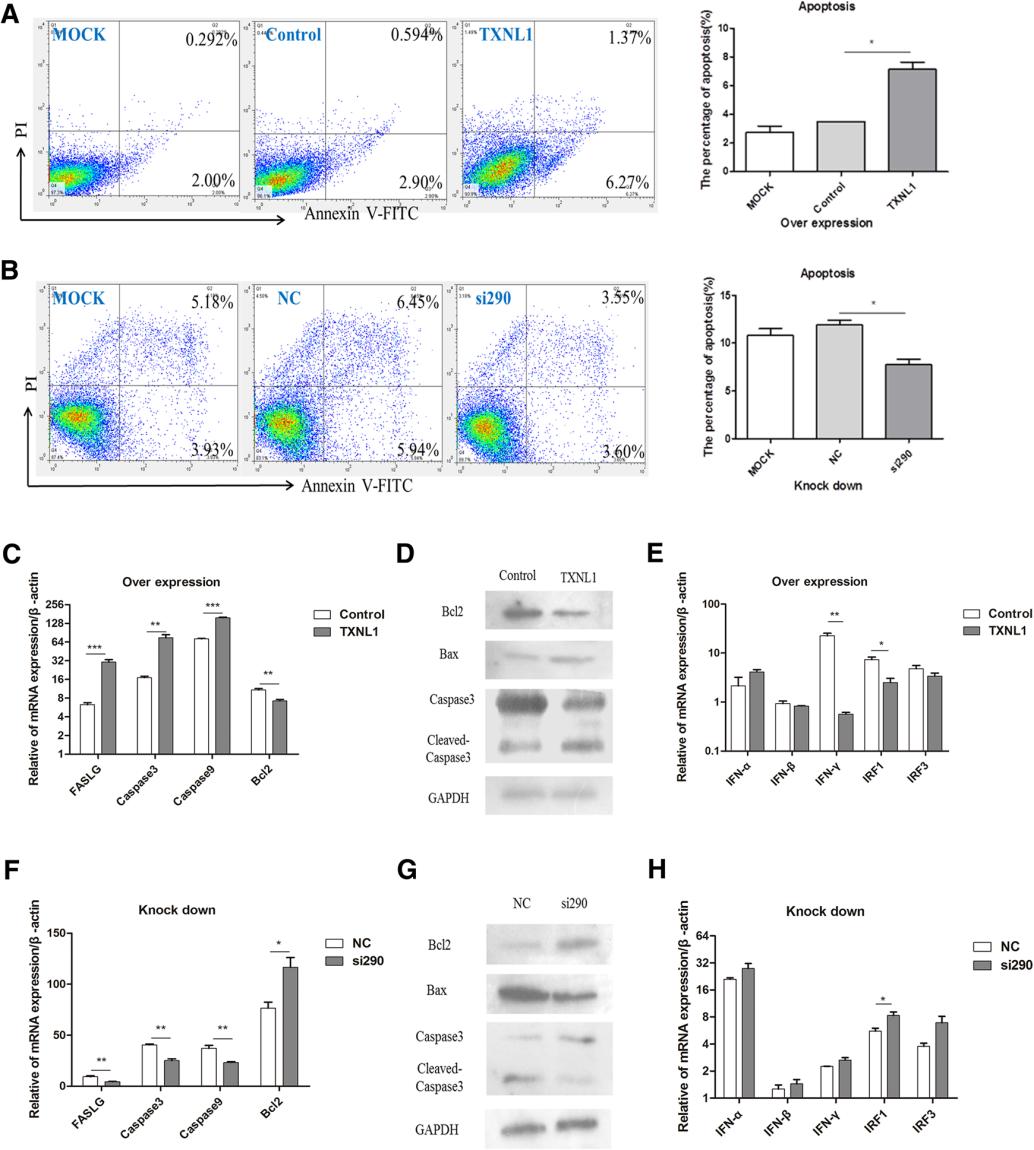
**TXNL1 induces apoptosis in DF-1 cells through the Bcl-2\Bax-Caspase-3 pathway. A** Dot plot showing the flow cytometric analysis of phosphatidylserine (PS) translocation after staining with annexin V and PI in mock, control (pCMV-HA), and TXNL1-transfected DF-1 cells at 48 h. A representative of three independent experiments is shown. The lower right quadrant represents the early apoptotic cells (annexin v-positive), while the upper right quadrant shows the late apoptotic and necrotic cell populations (annexin v and PI-positive). **B** Dot plot showing the flow cytometric analysis of PS translocation after staining with annexin V and PI in mock, NC, and si290-transfected DF-1 cells at 36 h. **C** pCMV-HA-TXNL1 and control plasmids were transfected into DF-1 cells for 48 h before the cells were harvested. The RNA was extracted according to the previously described method. The mRNA level of some apoptosis-related genes (proapoptosis genes Caspase-3, Caspase-9, and FasLG and the antiapoptosis gene Bcl-2) were detected using Q-PCR. **D** DF-1 cells were transfected with pCMV-HA-TXNL1 and control plasmids for 48 h. Whole-cell extracts were prepared for Western blot analysis that was specific for the proteins indicated. **E** TXNL1 and control were transfected into DF-1 cells for 48 h before the cells were harvested. The mRNA levels of some interferon-related genes (IFN-α, IFN-β, IFN-γ, IRF1, IRF3) were detected using Q-PCR. **F** Si290 and NC were transfected into DF-1 cells for 36 h before the cells were harvested. The mRNA level of some apoptosis-related genes were detected using Q-PCR. **G** DF-1 cells were transfected with si290 and NC for 36 h. Whole-cell extracts were prepared for Western blot analysis that was specific for the indicated proteins. **H** si290 and NC were transfected into DF-1 cells for 36 h before the cells were harvested. The mRNA levels of some interferon-related genes were detected using Q-PCR.

The V protein is known to inhibit apoptosis through the Bcl-2\Bax-Caspase-3 pathway in DF-1 cells. This study explored whether TXNL1 was related to apoptosis in DF-1 cells. Flow cytometric analysis revealed that both the early and late apoptotic cells were found to be enhanced in TXNL1-transfected DF-1 cells when compared to empty vector cells at 48 h (Figure 4A). Furthermore, to determine the involvement of the intrinsic pathways of apoptosis in TXNL1-induced apoptosis, the activation of a series of apoptosis-related genes at the mRNA level was assessed. The result revealed that the expression levels of the proapoptotic genes (Caspase-3, Caspase-9 and FasLG) were all significantly higher in TXNL1-transfected DF-1 cells than those in the control (pCMV-HA), while the expression level of the antiapoptosis gene (Bcl-2) was lower in TXNL1-transfected cells than that in the control (Figure 4C). To further verify the results, we evaluated the expression of apoptosis-related genes at the protein level. The figure shows that when TXNL1 was overexpressed in DF-1 cells, the expression levels of cleaved Caspase-3 and Bax were significantly higher than those of the control, while the expression of Bcl-2 was lower than that of the control (Figure 4D). These data show that the expression of TXNL1 was able to induce apoptosis in DF-1 cells through the Bcl-2\Bax-Caspase-3 pathway. To further confirm whether TXNL1 directly plays a proapoptosis role in the Bcl-2\Bax-Caspase-3 pathway, the endogenous TXNL1 was knocked down, and the abovementioned experiments were repeated. From the picture, it is clear that both the early and late apoptotic cells were reduced in DF-1 cells compared to those in the NC and mock groups at 36 h (Figure 4B). A series of apoptosis-related genes at the mRNA and protein levels were tested, and knockdown of the endogenous TXNL1 produced the opposite results to those that occurred when TXNL1 was overexpressed (Figures 4F and 4G).

To confirm whether TXNL1 induces apoptosis through the IFN pathway, a series of IFN-related genes were tested at the mRNA level. Interferon regulatory factor 1 (IRF1) serves as an activator of genes involved in immune responses. In this work, overexpression of TXNL1 downregulated IRF1 expression but did not affect the expression of IFN-α/β and IRF3 (Figures 4E and H), suggesting that the IFN-α/β and IRF3 pathway may not be involved in the effect of TXNL1 on apoptosis. Furthermore, the mRNA of IFN-γ was downregulated by overexpression of TXNL1 in DF-1 cells, indicating that IFN-γ did not play a major role in TXNL1-induced apoptosis in DF-1 cells.

## Discussion

The V protein of paramyxovirus can interact with MDA5 (melanoma differentiation-associated gene 5) and inhibit the production of IFN-β by preventing the activation of both NF-κB and IRF-3 [26, 27]. To further explore the function of the V protein by investigating its interactions with host proteins, it has been found using a Y2H technique that the V protein can interact with TXNL1 (Figure 1A). As an intracellular antioxidative protein, TXNL1 can regulate the reduction of reactive oxygen species [28, 29]. Among all NDV structural proteins, only the matrix (M) protein was reported to be located in the nucleus [30]. Between the two nonstructural proteins of NDV, there have been no reports of the subcellular localization of the V protein. Here, the results first suggest that the V protein may be located in both the cytoplasm and nucleus in DF-1 cells (Figure 1B). TXNL1 staining has been reported to be localized in the cytoplasm and nucleus [20]. In the present study, TXNL1 was found in the cytoplasm in DF-1 cells (Figure 1B), which is consistent with the localization of the V protein. Based on these results, it may be suggested that the V protein can regulate the function of TXNL1 in the cytoplasm and nucleus, but this hypothesis needs to be further verified. Several recent studies have shown that the V protein downregulates the expression of TXNL1 at the mRNA and protein levels in DF-1 cells (Figures 1C and 1D), suggesting that the V protein may exert its function through TXNL1.

The V protein of NDV has an antiapoptotic function [12]. However, the potential regulatory mechanism involved is still unclear. Here, to study how the V protein inhibits apoptosis, the antiapoptotic mechanism of the V protein was investigated. The Q-PCR and Western blot results suggest that the V protein inhibited apoptosis in DF-1 cells by targeting Bcl-2, Bax and Caspase-3, and this result suggests that the V protein could induce NDV proliferation by inhibiting programmed cell death, which may be an important mechanism by which the V protein facilitates viral replication.

The V protein has been reported to promote NDV replication by inhibiting the generation of interferon [10, 31]. However, whether that function is accomplished through the downregulation of TXNL1 is unclear. To verify this hypothesis, the effects on NDV replication were examined in DF-1 cells with overexpressed and knocked down TXNL1 cells. The results show that the overexpression of TXNL1 inhibits viral replication (Figures 3B and C) and that the knockdown of endogenous TXNL1 promotes viral replication (Figures 3E and F). These results suggest that the V protein promotes NDV replication by downregulating the expression of TXNL1. Furthermore, TXNL1 can resist cisplatin by interacting with X-ray repair cross complementing group 1 (XRCC1) in human gastric cancer [20]. TXNL1 has been reported to induce apoptosis by downregulating Bcl-2 in gastric cancer cells [21]. To further explore how TXNL1 affects viral replication, the role of TXNL1 in apoptosis signaling pathways was first tested. The results show that TXNL1 promotes apoptosis in DF-1 cells by targeting the Bcl-2\Bax-Caspase-3 pathway.

IFN-β could induce caspase-dependent apoptosis and necroptosis in cancer cells [32]. However, in this work, the mRNA of IFN-β was not significantly changed by either overexpression or knockdown of TXNL1 in DF-1 cells (Figures 4E and H), indicating that TXNL1 regulates cell apoptosis through a pathway other than the interferon pathway. It is suspected that some unknown mechanisms, other than inducing cell apoptosis, may be involved in the effect of TXNL1 on the suppression of NDV replication.

**Figure 5:**
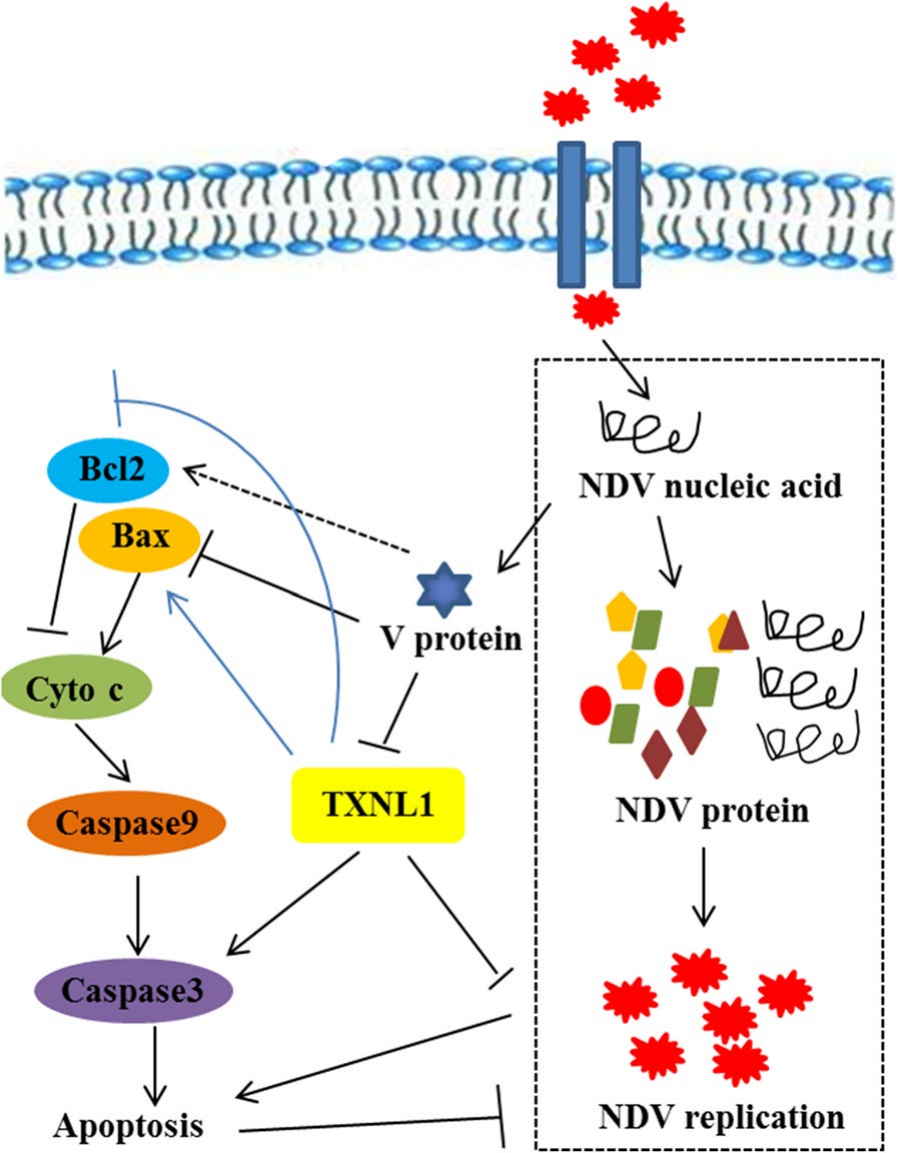
**Model of NDV V protein regulating apoptosis in DF-1 cells.** When NDV invaded the DF-1 cells, the virus replicated and translated a number of V proteins. V protein inhibits the activation of Bax and activates Bcl-2, thereby inhibiting apoptosis. TXNL1 inhibits the activation of Bcl-2 and activates the Bax-Caspase-3 pathway, thereby promoting cell apoptosis. At the same time, the V protein inhibits the expression of TXNL1. In this process, apoptosis inhibits the replication of NDV.

In summary, the research findings revealed the mechanism of the antiapoptotic function of the V protein (Figure 5). The V protein inhibits apoptosis by downregulating TXNL1 in DF-1 cells, which can provide new avenues for the further study of the V protein and can provide information about the interactive relationships between viral genes and host proteins. However, further studies need to be performed to confirm whether other apoptotic pathways and host proteins are involved in the antiapoptotic function of the V protein in DF-1 cells. These findings should help to further the understanding of how NDV can induce apoptosis and provide ideas for further research.
